# Vaccination in Leishmaniasis: A Review Article

**DOI:** 10.52547/ibj.26.1.35

**Published:** 2021-12-20

**Authors:** Latifeh Abdellahi, Fariba Iraji, Anahita Mahmoudabadi, Seyed Hossein Hejazi

**Affiliations:** 1 Skin Diseases and Leishmaniasis Research Center, Isfahan University of Medical Sciences, Isfahan, Iran;; 2 Department of Dermatology, School of Medicine, Isfahan University of Medical Sciences, Isfahan, Iran;; 3 School of Medicine, Augusta University, UGA Partnership, Athens,Georgia, United States of America;; 4 Skin Diseases and Leishmaniasis Research Center, Department of Parasitology and Mycology, School of Medicine, Isfahan University of Medical Sciences, Isfahan, Iran

**Keywords:** Immune response, Leishmaniasis, Vaccination

## Abstract

Leishmaniasis is caused by protozoan Leishmania parasites that are transmitted through female sandfly bites. The disease is predominantly endemic to the tropics and semi-tropics and has been reported in more than 98 countries. Due to the side effects of anti-Leishmania drugs and the emergence of drug-resistant isolates, there is currently no encouraging prospect of introducing an effective therapy for the disease. Hence, it seems that the key to disease control management is the introduction of an effective vaccine, particularly against its cutaneous form. Advances in understanding underlying immune mechanisms are feasibale using a variety of candidate antigens, including attenuated live parasites, crude antigens, pure or recombinant Leishmania proteins, Leishmania genes encoding protective proteins, as well as immune system activators from the saliva of parasite vectors. However, there is still no vaccine against different types of human leishmaniasis. In this study, we review the works conducted or being performed in this field.

## INTRODUCTION

Leishmaniasis is a vector-borne disease caused by more than 30 species of *Leishmania* parasites. The disease has a broad clinical picture, ranging from skin lesions to fatal visceral infections^[^^1^^].^ Leishmaniasis is endemic to four continents and more than 98 countries^[^^2^^]^. According to the WHO, 350 million people are at risk for leishmaniasis^[^^2^^]^. Leishmaniasis is found in humans in two main forms: CL and VL. Approximately 58,000 VL cases and 220,000 CL cases are reported annually^[^^2^^]^. The CL is divided into cutaneous, mucocutaneous, and diffused cutaneous types^[^^2^^]^. *L.* *tropica* and *L. major* are the main causes of CL, while *L. infantum* and *L. donovani* are the main causes of VL. Different species of rodents in various parts of Iran act as a reservoir for rural CL. These species include *Rhombomys opimus* and *Meriones libycus* found in the central and northeast, *M. libycus*, *M. persicus*, and *M. hurrianae* in the south, as well as *Tatera* *indica* and *Nesokia indica* in the west and southwest^[^^3^^,^^4^^]^. The *Leishmania* parasite is transmitted in the Old World, including Europe, Africa, and Asia, by the bite of the female sandfly of the genus *Phlebotomus*, and in the New World, including America, by *Lutzomyia*. The main hosts are vertebrates, and the most commonly infected hosts include humans, dogs, and rodents^[^^5^^]^. The sandfly family consists of five genera and 700 species, of which about 30 species are involved in the transmission of the *Leishmania* parasite^[^^6^^]^. Table 1 shows the main species of *Leishmania* that cause human disease. Over the years, many types of research have been conducted on the *Leishmania *vaccine. In each of these studies, candidate antigens were produced using improved laboratory techniques and various experimental models were examined. An overview of the results from the past to the present investigations can provide a fruitful research strategy for researchers. Meanwhile, such studies have shown that different vaccine administration routes can affect protective immunity. Despite the large number of preclinical vaccine candidates, and approaches designed to emulate this protective response^[^^7^^]^, the successful transition of *Leishmania* vaccines into human trials has remained elusive, though considerable efforts are underway^[^^8^^,^^9^^]^. Therefore, the purpose of this article is to provide a more comprehensive review of the current advances in leishmania vaccine development. 

**Immunity against leishmaniasis**

Macrophages are the primary hosts for *Leishmania*, but their role in preventing or progressing the disease has been described in T-cell-dependent behavior; however, the fate of the infected macrophages before T cell presence is not well-known^[^^10^^]^. Because specileilized T cells apeare late in the infection, the parasite is able to regulate disease progression in the host. 

Parasites can manipulate killing mechanisms of macrophages, at the time of their entry, and stimulate the production of IL-4 and certain disease-stimulating factors by T cells, leading to the progress of the disease and survival of the parasite^[^^11^^]^. As soon as the parasite diverts the CD40 signaling pathway to the pre-parasitic pathway in macrophages, the interaction between the CD40 ligand presented on activated T cells surfaces and CD40 receptors of infected macrophages cannot activate the anti-parasitic pathway, and probably reaction of T cell-macrophage does not maintain the host^[^^12^^]^. In addition to the host apoptosis, stimulation of parasite apoptosis can be one of the therapeutic goals to increase the effectiveness of antiparasitic drugs. For instance, the study of Sengupta

*et al.*^[^^13^^]^ showed that the natural indoloquinoline alkaloid cryptolepine causes a decrease in the cell viability of *L.* *donovani* AG83 promastigotes in both time- and concentration-dependent manners by increasing ROS and lipid peroxidation production and decreasing cellular glutathione levels. The results of Roy *et al.*'s^[^^14^^]^ study also indicated that the plant carbazole alkaloid exerts *in vitro* and *in vivo* antileishmanial activity by the modulation of redox homeostasis. Furthermore, about inducing host apoptosis, researches have demonstrated the integration of expressional cassettes containing pro-apoptotic genes in *Leishmania* by transgenic method or downregulating antiapoptotic molecule by miRNA could accelerate the apoptosis process of infected macrophages, restrict the possibility of differentiation and induce more proliferation of *Leishmania*. These events would result in the expansion of the disease, and the appearance of the lesion^[^^15^^]^. A study by Aghaei *et al*.^[^^16^^]^ signified that the transgenic *L.* *infantum* expressing mLLO-BAX-SMAC proteins can accelerate the apoptosis of infected macrophages compared to wild-type *Leishmania*. It means that transgenic Leishmania is proved to increase the rate of apoptosis in infected macrophages compared to intact strain. Since metacaspases are the key regulators of death or life of parasites, and these proteins do not exist in mammals, they can be considered as targets for fighting against parasitic infections in the future^[^^17^^]^.

**Table 1:** The main species of *Leishmania* that cause human disease

***Leishmania*** ** species**	**Disease form in humans**	**Geographical distribution**	**Reservoir**	**Vectors**
*Leishmania aethiopica* ^ *^	Localized CL,	Ethiopia, Kenya	Rock hyraxes	*P. longipes*
	Diffuse CL			*P. pedifer*
*L. major* ^*^	Localized CL	North Africa, the Middle East and Central Asia, Sub- Saharan Africa and Sahel belt, Sudan, North India, and Pakistan	Rodents	*P. papatasi* and *P. duboscqi*
*L. mexicana* ^**^	Localized CL	Central America	Forest rodents	*Lutzomyia olmeca*
*L.amazonensis* ^**^	Localized CL	South America, north of the Amazon	Forest rodents	*L. flaviscutellata*
				
*L.braziliensis* ^**^	Localized CL	South America,	Forest rodents,	*Psychodopygus*
	Mucocutaneous leishmaniasis	Central America and Mexico	peridomestic animals	*Lutzomyia spp.*
*L. peruviana* ^**^	Localized CL	West Andes of Peru., Argentine highlands	Dog	*L. verrucarurn*, *L. pvmenis*
*L. infantum* ^*^	VL Localized CL	Mediterranean basin; Middle East and Central Asia to Pakistan; China; Central and South America, southern Europe, northwest Africa	Dogs, cats, foxes, and jackals	*P. perniciosus *and *P. arias*
*L. donovani* ^*^	VL	Ethiopia, Sudan, Kenya, India, China, Bangladesh, Burma	Human anthroponosis, Rodents Sudan, canines	*Phiebotomus argentipes*, *P. orientalis*, and *Pseudostomatella martini*

^*^Old World species; ^**^New World species; *P.*, *Phlebotomus*; *L.*, *Lutzomyia*

**Vaccination concepts in leishmaniasis**

There are some facts to support the possibility of developing an effective vaccine against CL. However, due to the increased resistance to first-line drugs and the toxicity of second-line drugs, the development of an effective vaccine against the disease is very desirable. The use of vaccines is advantageous over chemotherapy as they induce long-lasting effects and can be administered both in prophylactic and therapeutic modes. Also, the vaccine will not counter the problem of resistance as in the case of chemotherapy^[^^18^^]^. As stated in a study published by Thomaz-Soccol *et al.*^[^^19^^] ^in 2018, the number of patents for leishmaniasis vaccines is 74 in the United States and 36 in Brazil. In Brazil, 20,000 cases of leishmaniasis and more than 3,000 cases of VL, and in India, 8,000 cases of VL are reported annually^[^^20^^]^. Spain and France are still endemic for VL. In France, for example, the prevalence of VL is 0.22 per 100,000 population in the endemic regions^[^^21^^]^. Therefore, vaccination against leishmaniasis is essential in these areas. Moreover, the highest number of patents was reported in that study to be related to the private sector (94 cases), and the lowest was related to cooperation between universities and companies (11 cases); however, universities and noneducational public institutions had 65 and 13 patent cases, respectively^[^^21^^]^. Therefore, the need for more cooperation between public and private institutions seems to be necessary.

**Challenges of efficient vaccine design**

To date, many attempts have been made to test clinically prepared vaccines in various human trials, but they have been ineffective. It is widely believed that this problem arises from economic and financial pressures^[^^22^^]^. Some studies have shown that using the whole parasite leads to inefficient antigen presentation and anti-*Leishmania* memory cell development, thus reducing immunity^[^^23^^-^^25^^]^. Also, preserving central memory T cells does not require the presence of parasites^[^^26^^]^. There may not have been a suitable human adjuvant system for testing these vaccines^[^^27^^-^^29^^]^. Vaccination provides long-term protection in the absence of attenuated strains such as LdCEN^-/- ^(centrin mutant) or PMMΔ (phosphomannosemutase). This finding was performed in a mouse model and not in humans. Injection of protective antigens in different models or immunotherapy has helped to find the factors involved in increasing anti-*Leishmania* immunity. One of the major problems facing the vaccine against CL is the fact that despite causing cutaneous disease, the Old and New World parasites, *L. major *and *L. mexicana/L. amazonensis*, respectively, are significantly different^[^^30^^]^. There are differences in virulence factors between these species, as well as in the immune responses induced by them. For instance, LPG is a virulence factor for *L. major*^[^^31^^]^, but not for *L. Mexicana*^[^^32^^]^. During *L. major *infection, the protective role of Th1 responses has been established, but *L. amazonensis* can persist in the presence of Th1 responses and cause minimal disease in the complete absence of T cells^[^^33^^]^. These findings show major, but not well-understood, differences in the immunobiology of parasites that appear to cause the same disease. This matter may have implications for the vaccine development process as the anti-CL vaccine may have different needs for the Old and New World leishmaniases. Therefore, a vaccine against CL caused by *L. major* might not necessarily be effective against the New World spectrum of diseases, including mucocutaneous and diffuse cutaneous forms. Another challenge for the vaccine is to obtain protection against VL even if it is efficacious against varied forms of CL.

**Immunization methods against CL**

***Leishmanization***

Adler observed that Lebanese children whose arms have been exposed to infected mosquitoes by their mothers will be protected against severe forms of the disease in the future^[^^34^^]^. This process was not followed because it caused uncontrolled growth of skin lesions and also led to a high prevalence of the disease in people with suppressed immune systems, particularly those with HIV and organ transplants^[^^35^^,^^36^^]^. The first method of immunization against leishmaniasis known as "leishmanization” was developed in 1940 and has been used in various countries for several years^[^^37^^]^. This vaccine was discontinued due to its lack of safety and is now limited to the vaccine registered in Uzbekistan and the vaccine used in clinical trials in Iran^[^^38^^,^^39^^]^. In this procedure, live and active *L. major* promastigotes are injected intradermally into the anatomical position of the deltoid muscle. An active ulcer then develops and eventually heals on its own. The result of this method is long-term immunity against rural and urban leishmaniasis. Tables 2 and 3 shows leishmanization experiments in Iran and USSR countries.

**First-generation vaccines**

These vaccines contain the whole body of the parasite with or without adjuvant^[^^39^^]^. First-generation vaccines replaced leishmanization, and the vaccine is now used in some human trials. These categories include killed, live attenuated, and fractionated vaccines^[^^40^^]^. Table 4 lists the first-generation vaccines with full specifications. 

***Killed vaccines***

This type of vaccine was developed and evaluated by Mayrink *et al.* in Brazil^[^^41^^,^^42^^]^. The result of the leishmanin skin test was satisfactory, but the vaccine had only a 50% protective effect. In Venezuela, Sharples *et al.*^[^^43^^] ^ used a mixture of killed *L. amazonensis*, *L. mexicana*, and *Bacillus Calmet Guerin* to treat CL, resulting in a 95% improvement and activation of Th1 immunity^[^^43^^-^^45^^]^. Studies in Brazil have shown that a mixture of killed *L. amazonensis* with half a dose of meglumine antimoniate is very effective in treating CL^[^^46^^]^. According to a study conducted in Ecuador, a proportion of *L. brasiliensis*, *L. guianensis*, and *L. amazonensis* provided favorable protection against CL^[^^47^^-^^49^^]^. Two studies in Iran have shown that autoclaved *L. major* vaccine with BCG is safe but does not provide promising immunity against CL^[^^50^^,^^51^^]^. The results of a study by Mahmoodi *et al.*^[^^52^^]^ revealed that cases who received the ALM + BCG vaccine had a higher stimulation index and IFN-γ levels than those who received BCG alone or in the control group. The results of this study showed that the induction of Th1 immune response in volunteers who received the vaccine was much lower than those with or without a previous history of leishmaniasis, and it was assumed that these individuals became immune^[^^52^^]^. Th1 is activated in *L. major* infection, but *L. amazonsensis* can remain active in the presence of Th1 and can reduce the T cell response. Therefore, the vaccine made for *L. major* is neither effective for another leishmaniasis nor VL. In general, vaccination with killed *Leishmania *promastigotes could be considered as a safe and economical treatment; nevertheless, further trials aiming at the evaluation of different adjuvants potentially pave the way for more efficient vaccines^[^^53^^]^.

***Live attenuated vaccine***

These vaccines are currently the gold standard. In attenuated live vaccines, the parasite is both nonpathogenic and superior to killed vaccines^[^^54^^]^. Methods of preparing attenuated live parasites include long-term *in vitro* culture^[^^55^^]^, use of temperature sensitivity^[^^56^^]^, gamma radiation^[^^57^^]^, chemical mutagenesis^[^^58^^]^, and culture with gentamicin^[^^59^^]^. Titus and co-workers^[^^60^^] ^developed a live attenuated vaccine by knocking down certain *Leishmania* genes. Examples in this regard are the *DHFR-TS*^[^^60^^]^ and the *lp2* gene, which encodes an enzyme, transports guanosine diphosphate mannose to the Golgi apparatus^[^^61^^-^^63^^]^, the *lpg2* mutant from *L. mexicana*^[^^64^^]^, the CP (*cpa* and *cpb*) from *L. mexicana*^[^^65^^,^^66^^]^, the *SIR2* from *L. infantum*^[^^67^^]^, and the BT1 gene from *L. donovani*^[^^68^^]^.

***Suicidal cassettes***

 Muyombwe *et al.*^[^^69^^] ^followed a method of producing a vaccine against leishmaniasis, which was to induce suicide genes. This method is performed by inducing drug-sensitive genes. They used a combination of thymidine kinase and gancyclovir against *L. major* and finally using gancyclovir treatment, partial to complete protection was achieved^[^^70^^,^^71^^]^. Besides, the susceptible strain of *L. major*, which contained the altered thymidine kinase *HSV-1* (*tk*) gene and the cytosine deaminase gene from *Saccharomyces cerevisiae* (*cd*), increased susceptibility to gancyclovir and 5-fluorocytosine. *L. major* infection recovered within two weeks of treatment with either drug alone or in combination with ganciclovir and 5-fluorocytosine^[^^70^^,^^71^^]^.

**Fractionated vaccine**

This kind of vaccine is advantageous due to its high purity and yield. Several molecules, either membrane proteins, such as HASPB1 and A2 protein, or soluble fractions of the parasite, i.e. PDI, TPI, elF-2, aldolase, enolase, P45, tryhpanothione reductase, and recombinant F14, among others have been used as a potential target for vaccination, both against cutaneous and VL. Also, some polyproteins have been tested with some degrees of success (Q protein, Leish-111f, 110f etc.)^[^^72^^]^.

**Table 2:** Leishmanization experiments in Iran

**Year**	**Study place**	**No.of individuals**	***Leishmania*** ** species**	**Infected with disease (%)**	**Comment**	**Ref.**
1946	Tehran	120	*L. tropica major*	90	Cross protection against *L.tropica minor*	
1977	Isfahan	250	*L. major*	47	The incidence rate of CL in leishmanized children was one-sixth to one-seventh to control group.	^112^^,^^113^
1982-86	Isfahan and Dezful	160,000	*L. major*	89.5	Under 1% of new cases of CL were among leishmanized people.	^112^^,^^113^
1983-1989	On army recruits and revolutionary guard	1800,000 and 6000 refugees	*L. major*	56.7–90	Reduction of the incidence rate of CL by Leishmanization among leishmanized people between one-sixth to one-eighth of its original level	^113^^,^^114^
2005	Tehran	28	*L.major*	100	Total protection was seen in 100% (11/11) of volunteers.	
1989	Individuals receiving NLCV (no. 27)	unvaccinated individuals (no.30)	*L. major*	61.5% in vaccinated and 90% in unvaccinated individuals	With 27% protection in the NLCV group	
2001	Isfahan Province	200	Deep-freeze promastigote forms of *L. major*	40–45	Production of *L. major* under good manufacturing practices condition at Razi Institute	^unpublished^

NLCV, nonliving crud vaccine

**Second-generation vaccines**

Second-generation vaccines are based on synthetic or recombinant subunits and genetically modified *Leishmania* strains, recombinant bacteria, or viruses carrying *Leishmania* antigen genes^[^^73^^-^^75^^]^. A summary of these vaccines against *Leishmania* is given in Table 5.

**Vaccines based on nonpathogenic** ***Leishmania***

In 2015, Katebi *et al.*^[^^76^^] ^showed that vaccination with *L. tarentolae*-PpSP15 in combination with CpG as a prime-boost modality confers strong protection against *L. major* infection, which was superior to other vaccination methods discussed in the present study. This approach represents a novel and promising strategy for vaccination against Old World CL. In 2014, Zahedifard *et al.*^[^^77^^]^ demonstrated the effect of a novel combination of protective parasitic antigens created by *L. tarentolae*, together with sandfly salivary antigen as a vaccine strategy against *L. major* infection. The immunogenicity and protective effect of different DNA/Live and Live/Live prime-boost vaccination with live *L. tarentolae* expressing CPs (type I and II, CPA/CPB) and PpSP15 from *Phlebotomus papatasi*, were tested in BALB/c and C57BL/6 mice. Both humoral and cellular immune responses were assessed before challenge and at 3 and 10 weeks after *Leishmania*  infection. In both strains of mice, the strongest protective effect was observed when the mice primed with PpSP15 DNA and then received PpSP15 DNA and live recombinant *L. tarentolae *as a booster^[^^77^^]^. In 2015, Shahbazi *et al.*^[^^78^^]^ vaccinated outbreed dogs with a prime-boost regimen based on recombinant *L*. *tarentolae *expressing the *L*. *donovani *A2 antigen, along with CP genes (CPA and CPB^-CTE^) and evaluated its immunogenicity and protective immunity against *L*. *infantum* infectious challenges. 

**Table 3:** Early leishmanization experiments in USSR countries

**Year**	**Inoculum**	**Number**	**Infected with disease (%)**	**Comment**	**Ref.**
1942–1968	1.5 × 10^6^	647	60–90	Used infected hamster tissue	
1972	10^6^	65	100	A new isolate replaced older ineffective strain	
1978	2 × 10^6^	475	14-100	High level of nodules	
1979	4 × 10^6^	39	100	Pretest of frozen vaccine	
1968	0.8 × 10^6^	2245	98	93.2% of ulcers <2 cm at 2 months	
1968	0.1–1.2 × 10^6^	12500	90	Found little influence of culture age, medium or number	
2018	-	9500	96-100	-	^1^ ^18^

They showed that vaccinated animals produced significantly higher levels of IgG2, but not IgG1, as well as IFN-γ and TNF-α, but low IL-10 levels, before and after challenge as compared to control animals. Protection in dogs was also associated with a strong DTH response and a low parasite burden in the vaccinated group. Overall, immunization with recombinant *L*. *tarentolae *A2-CPA-CPB^-CTE^ proved to be immunogenic and induced partial protection in dogs, hence representing a promising live vaccine candidate against canine VL^[^^78^^]^. In 2013, Saljoughian *et al.*^[^^79^^]^ used a tri- gene fusion recombinant *L. tarentolae* expressing the *L. donovani* A2 antigen, along with CPs, as a live vaccine. Their results showed that immunization with both prime-boost A2-CPA-CPB^-CTE^-recombinant *L. tarentolae* protects BALB/c mice against *L. infantum* challenge. This protective immunity is associated with the Th1 immune response due to the high levels of IFN-γ production before the challenge, leading to a significant increase in the IFN-γ/IL-10 ratio compared to the control groups. In addition, this immunization induced an elevated level of IgG1 and IgG2a humoral immune responses. Protection in mice was also associated with a high NO production and low parasite burden. Altogether, these results indicate the potential of the A2-CPA-CPB^-CTE^-recombinant *L. tarentolae* as a safe live vaccine candidate against VL^[^^79^^]^.

***Lactococcus lactis*** **as a tool for** ***Leishmania*** **vaccination**

*L. lactis *is a well-defined, food-grade lactic acid bacterium commonly known as generally recognized as safe status. A better understanding of this bacterium at a molecular level has led to the development of unprecedented genetic tools that enable the expression of heterologous proteins. Consequently, the ability of *L. lactis *to express and deliver these proteins to eukaryotic hosts offers a promising approach to achieve potent treatments for various diseases. Currently, 13 genera have been classified under the lactic acid bacterium group, including *Lactococcus*,* Lactobacillus*,* Streptococcus*,* Pediococcus*,* Para-lactobacillus*,* Enterococcus*,* Carnobacterium*,* Lacto-sphaera*, *Leuconostoc*,* Oenococcus*,* Tetragenococcus*,* Weisella*,* and *Vagococcus^[^^80^^]^. In 2012, Hugentobler *et al.*^[^^81^^]^ described the generation of *L. lactis*(*alr*-) strain as the vector expression of the protective *Leishmania* antigen, LACK, in the cytoplasm, secreted or anchored to the bacterial cell wall or co-expressing mouse IL-12. They showed that oral immunization using live *L. lactis*, secreting both LACK and IL-12, was the only regimen that partially protected BALB/c mice against the next *L. major* challenge. This issue highlights the importance of temporal and physical proximity of the delivered antigen and adjuvant for optimal immune priming by oral immunization. In 2019, Torkashvand *et al.*^[^^82^^] ^expressed F1S1 fusion protein, including the N-terminal region of S1 subunit of PT and FHA type1 immunodominant domain by *L. lactis,* and evaluated its immunogenicity. Based on their results, mice immunized with LL-F1S1 produced significant levels of specific IFN-γ compared to controls and DTaP-immunized mice. The F1S1-specific IgG antibody response was lower in LLF1S1-immunized mice, while the IgG2a/IgG1 ratio was higher in this group compared to the DTaP-immunized mice. In 2020, Davarpanah and co-workers^[^^83^^]^ explained that PpSP15 is an immunogenic salivary protein from *P. papatasi*. Immunization with *Lactococcus lactis* expressing sand fly PpSP15 salivary protein has been shown to protect against *L. major* infection. In their study, BALB/c mice were challenged with *L*. *major* plus *P*. *papatasi* salivary gland homogenate. Evaluation of footpad thickness and parasite burden displayed a delay in disease development and reduced the number of parasites in PpSP15 vaccinated animals as compared to the control group. In addition, vaccinated mice exhibited Th1 type immune responses. Importantly, immunization with *L*. *lactis*-PpSP15-EGFP^cwa^ enhanced long-term memory in mice, which lasted for at least six months. 

**Table 4:** Types of first-generation vaccines against *Leishmania*

**Antigen**	**Vaccine form/ adjuvant/del. system**	**Animal model**	**Targeted disease (** ***Leishmania*** ** spp.)**	**Summary of the experimental system**	**Result**	**Another outcome**	**Ref.**
*L. major*	Pathogenic 10^4^ live promastigotes	C57BL/6	CL/*L. major*	Immunized through the ear (i.d.) and footpad (s.c.). Challenged 7 weeks later with 10^3^ promastigotes	Protection	s.c. route more effective enhanced IFN-γ and IL-10 levels in s.c. and i.d. immunization, respectively.	
							
*L. major*	Nonpathogenic live promastigotes	C57BL/6 BALB/c	CL/*L. major*	Immunized by intraperitoneal or subcutaneous injection. Challenged with pathogenic promastigotes	Protection	Complete protection in C57BL/6 mice while partial in BALB/c mice	
							
*L. braziliensis*	Avirulent *L. braziliensis*	BALB/c	CL/*L. major*	Immunization with Nmethyl- N′-methyl-N′- nitro-N-nitrosoguanidine treated promatigotes	Protection	Immunity conferred and transferred by Lyt-1+ cells	
							
*L. major*	γ-irradiated *L. major*	CBA	CL/*L. major*	Immunized through subcutaneous injection. Challenged with two strains *of L. major*	Protection	LN cells activated infected macrophages *in vitro* to kill the parasite	
							
*L. major*	LPG deficient avirulent *L. major*	BALB/c	CL/*L. major*	Vaccination with CD4^+^ T-cell line derived from avirulent promastigote immunized mice. Challenged with a virulent strain	Protection	Enhanced TNF and IL-2 production, suppressed IL-4, negative DTH	
							
*L. mexicana L. major*	Long-term culture of 5 × 10^6^ promastigotes with gentamycin	BALB/c	CL/*L. mexicana/L. major*	Immunization with s.c. injection followed by challenge with 5 × 10^6^ wild type promastigotes	Protection	Lesion size reduced by 80%, signiﬁcantly reduced infected macrophages	
							
*L. donovani L. infantum*	Long-term culture of promastigotes with gentamycin	BALB/c	VL/*L. donovani/L. infantum*	Immunized subcutaneously followed by challenge with wild type promastigotes	Protection	Percentage of infected macrophages reduced by 91–99%	
							
*L. chagasi*	Attenuated 10^7^ promastigotes	BALB/c	VL/*L. chagasi*	Challenge with virulent promastigotes	No protection	----	
							
*L. chagasi*	10^7^ live promastigotes	BALB/c	VL/*L. chagasi*	Immunization (s.c.) and challenged both with 10^7^ live promastigotes	Protection	88% parasite reduction, increased IFN-γ, IL-10, and IL-4 levels, low TGF-β level	
							
*L. chagasi*	10^2^ or 10^4^ live promastigotes	BALB/c	VL/*L. chagasi*	Immunization (s.c.) with 10^2^ or 10^4^ promastigotes and challenged with 10^7^ live promastigotes	Intermediate protection	No protection in 10^2^ doses, low IFN-γ, high TGF-β levels, no effect on IL-10 and IL-4 production as compared to control	
							
*L. major L. chagasi*	10^2^ and 10^7^ live promastigotes	BALB/c	VL/*L. chagasi*	Challenged with 10^6^ *L. chagasi* promastigotes	No protection	----	
							
*L. chagasi L. donovani L. major*	DHFR-TS knock-out Promastigotes	BALB/c	VL/*L. chagasi*	Challenged with 10^7^ virulent *L. chagasi*	No protection	A negligible amount of IFN-γ Release	
							
*L. major*	DHFR-TS knock-out promastigotes	BALB/c BALB/c (nu/nu) CBA/T6	CL/*L. major*	Immunization through s.c., i.v. and i.m. routes. Challenged with 10^6^ virulent promastigotes	Protection	i.v. route, parasite burden reduced by158–1990 fold in BALB/c mice, i.m. and s.c. the route also produces protection in CBA mice but not in BALB/c mice.	
							
*L. major*	DHFR-TS knock-out promastigotes 10^4^, 10^6^, and 10^8^ dose	BALB/c C57BL/6	CL/*L. amazonensis*	Immunization through i.v. and s.c. routes	Partial protection	10^8^ dose developed 40–75% and 49–57% smaller lesion size in BALB/c and C57BL/6 mice, respectively	
							
*L. major*	DHFR-TS knock-out 10^8^ promastigotes	Monkey	CL/*L. major*	Immunization subcutaneously and challenged with 10^7^ promastigotes	No protection	Positive proliferative response (79%), no IFN-γ production, negative DTH response	
*L. major*	Live promastigotes Different doses	BALB/c	CL/*L. major*	Immunization with 10^6^, 3 × 10^3^, 10^3^, 3.3 × 10^2^, 1.1 × 10^2^, or 3.7 × 10^1^ dose. Challenge with 10^6 ^promastigotes	Protection only in 1.1 × 10^2^ Borderline disease in half of the 3 × 10^3^ dose no protection in other doses	Enhanced IFN-γ production with low IgG1/IgG2a ratio in protected mice, Th1/Th2 response (both IFN-γ and IL-4 levels high) in borderline disease mice, and Th2 response in progressive disease mice.	
							
*L. major*	lpg2−mutant promastigotes	BALB/c	CL/*L. major*	Immunization (s.c.) with 5 × 10^6^ promastigotes and challenged with wild type 2 × 10^6^ parasites	Protection	Suppressed IL-10 and IL-4 production, low IFN-γ level, negative DTH response	
							
*L. major*	Δlpg2−mutant promastigotes + CpG oligonucleotides	C57BL/6	CL/*L. major*	Immunization with Δlpg2 with a single dose of CpG ODN (50 μg)	Protection	100 fold parasite reduction, no IFN-γ production, no DTH response	
							
*L. mexicana*	CP mutant promastigotes	BALB/c C57BL/6 CBA/Ca	CL/*L. mexicana*	Immunization (s.c.) with 5 × 10^6^ Δcpa or Δcpb or both. Challenged with 10^6^ wild type promastigotes	Protection	Increased IFN-γ and IL-2 levels with low IL-4, no difference in IL-5, IL-10, and IL-12 levels, high IgG2a/ IgG1 ratio	
							
*L. mexicana*	CP deﬁcient promastigote	Hamster	CL/*L. mexicana*	Immunization (i.d.) with 10^3 ^Δcpb or Δcpa/cpb promastigotes and challenged with wild type *L. mexicana*	Protection	High IFN-γ, no difference in IL-10 while TGF-β, IL-4, and IL-12 p40 not detected	
							
*L. infantum*	SIR2 deﬁcient	BALB/c	VL/*L. infantum*	Immunization (i.p.) with 10^8^ promastigotes and challenged with 10^8^ wild type promastigotes	Protection	Enhanced NO level, high IFN-γ/IL-0 ratio, no difference in IL-4 and IL-2 levels, high IgG1 and IgG2a titer	
*L. donovani*	BT1 knock-out promastigotes	BALB/c	VL/*L. donovani*	Immunization (i.v.) with 5 × 10^7^ mutant promastigotes. Challenged with 5×10^7^ luciferase-expressing virulent promastigotes	Protection	Infection rate reduced by 75%, increased IFN-γ level, no IL-4 production	
							
*L. tarentolae*	Nonpathogenic *L. tarentolae* promastigotes	BALB/c	VL/*L. donovani*	Immunization (i.p.) with 5 × 10^6^ promastigotes and challenged with 5×10^7^ virulent *L. donovani* promastigotes	Protection	80-85% parasite reduction, enhanced IFN-γ production, no IL4, spleen cell proliferation increased by 17 fold	
							
*L. major*	Suicide system of promastigotes with thymidine kinase gene of HSV-1	BALB/c	CL/*L. major*	Mice infected by tk-transfected or wild type promastigotes and treatment given by ganciclovir	Partial to complete	-----	
							
*L. major*	tk-cd^+/+^ transfected promastigotes	BALB/c	CL/*L. major*	Mice infected with tk-cd^+/+^ transfected and wild-type promastigotes. Treatment is given by ganciclovir and 5-ﬂuorocytosine	Protection	Mice infected with transfected promastigotes were completely cured by either or both drugs.	
							
*L. amazonensis*	Porphyrogenic (DT) and non-porphyrogenic (ST) transfectants	Hamster	VL/*L. donovani*	Photodynamic vaccination with DT + ALA, DT - ALA, ST + ALA, or ALA. Challenged with 10^7^ amastigotes	Protection	99% parasite reduction, increased DTH, and lymphoproliferative response, high IFN-γ, iNOS, and IL-12 expression, high IgG2a titer	
							
*L. infantum*		Human and animal	CL	injecting one milliliter of the fraction intracutaneously in four different points of the skin. These were people who had been ill for at least three months	Protection		

**Table 5:** Second-generation vaccines against *Leishmania*

**Antigen**	**Adjuvant/delivery system**	**Animal model**	**Targeted disease (Leishmania spp.)**	**Result**	**Other outcomes**	**Ref.**
gp63	*S. typhimurium*	CBA, BALB/c	CL/*L. major*	Protection	Protection only in CBA mice, 67–78% parasite reduction, activated CD4+ T cells which secret IFN-γ and IL-2 but not IL-4, negative DTH response	
						
gp63	Alone and along with BCG or *C. parvum* or MDP	CBA, BALB/c	CL/*L. major*	Protection	Antigen alone reduced the lesion size comparable to those of gp63 + BCG, protection induced by gp63 + adjuvant varied depending on the site of vaccination relative to that of the challenge	
						
rgp63	*C. parvum*	BALB/c	CL.	No protection	----	
						
rgp63	*E. coli*	Monkeys	CL/*L. major*	Partial protection	Positive DTH response, no IFN-γ production, high IgG antibody level	
						
gp63	Liposomes liposomes + CFA	CBA	CL/*L. mexicana*	protection	The protection conferred only by gp63 + liposomes	
						
rgp63	*S. typhimurium*	BALB/c	CL/*L. major*	protection	Activated T cells secreted IFN-γ and IL-2 but not IL-4, high IgG2a levels, no IgG1, negative DTH response.	
						
rgp63	*S. typhimurium*	BALB/c	CL and VL*/L. major* or *L. donovani*	Protection	Protection induced against both species, high IFN-γ level, IL-2, and IL-4 not detectable, negative DTH response.	
						
rgp63	*S. typhimurium*	F1 (BALB/c C57BL/6)	CL/*L. mexicana*	Protection	High IFN-γ and IL-2 mRNA expression but not IL-4 and IL-10	
rgp63	Transfected BCG	BALB/c CBA/J	CL/*L. mexicana* or *L. major*	Protection	Protection against *L. mexicana* and *L. major *in both mouse strains, strong lymphoproliferative response.	^136^^,^^137^
						
gp63	Cationic liposomes	BALB/c	VL/*L. donovani*	Protection	86% and 81% parasite reduction in liver and spleen respectively, high IFN-γ and IgG2a levels even after challenge, low IL-4 production, positive DTH response.	
gp63 or rgp63	*E. coli*	Human	CL/VL	Protection	strong proliferative response to both species, high IFN-γ production in PBMC culture upon antigen stimulation.	
Peptide PT3 of gp63	Poloxamer or CFA or DC pulsed	BALB/c	CL/*L. major*	Protection	Protection only by PT3 (p154–168), enhanced IL-2 but not IL-4 production, no lesion in the second study while reduced lesion development in the third study	^141^^-^^144^
						
rgp63	Transfected L929 cells with CD40L + gp63	BALB/c C57BL/6	CL/*L. major* or *L. amazonensis*	Protection	Both strains of mice protected against both parasite species, high IL-12 production	
						
M-2	*C. parvum* Saponin CFA	CBA BALB/c C57BL/6	CL/*L. amazonensis*	Variable protection	*C. parvum* gave better results, followed by saponin, complete protection in CBA, partial in BALB/c, and no protection in C57BL/6, protection correlated with increased IgG1 and IgG2	
						
GP46/M-2	Vaccinia virus	BALB/c	CL/*L. amazonensis*	Protection	IL-2, IFN-γ, and IL-4 production, high IgG1, IgG2a, and IgM with low IgG3 and IgG2b	
						
PSA-2	*C. parvum*	C3H/He	CL/*L. major*	Protection	100-fold parasite reduction, predominant IgG1 with IgG2a and IgG2b before the challenge, high IFN-γ but no IL-4 level	
						
rPSA-2	Transfected *E. coli* + *C. parvum* ISCOM	C3H/He	CL/*L. major*	No protection	High IFN-γ production, high IgG1, IgG2a, IgG2b, and weak IgG3	
						
LACK/rp24	IL-12	BALB/c	CL/*L. major*	Protection	Upregulation of IFN-γ and downregulation of IL-4 transcripts	
						
rLACK	rIL-12	BALB/c	CL/*L. major*	Protection	Mice protected only when challenged after two weeks of last immunization, not protected when challenged after 12 weeks of immunization, high IFN-γ (after two weeks)	
						
rLACK	rIL-12	BALB/c	CL/*L. amazonensis*	Protection	After the challenge, the IFN-γ level decreased to the levels of IL-10 and IL-4, high anti-LACK and parasite-speciﬁc antibodies	
rLACK	-----	BALB/c	CL/*L. amazonensis*	No protection	A slight increase in IFN-γ level, IL-10, and IL-4 levels comparable to PBS control.	
						
FML	Saponin	BALB/c	VL/*L. donovani*	Protection	84.4% reduction in liver parasite burden, 79.1% and 89.1% increase in proliferative and antibody responses respectively, high antibody level.	
						
FML	Saponin	BALB/c	VL/*L. donovani*	Protection	94.7% liver parasite reduction, no change in IFN-γ level while signiﬁcant decrease in IL-10 production, high DTH response, increase in IgG, IgM, IgG1, IgG2a, and IgG2b anti-FML antibodies	
						
FML	Saponin	Swiss albino	VL/*L. donovani*	Protection	85.5% reduction in liver parasite burden, 80% increase in the antibody response	
						
FML	Saponin aluminum hydroxide	Swiss albino	VL/*L. donovani*	Protection	85% and 88% liver parasite reduction in FML + saponin and FML + Al(OH)3 group respectively, increased IgG2a level in the former group, similar IgG2b, and IgG3 in both vaccines	
						
FML	Saponin	Hamster	VL/*L. donovani*	Protection	Positive DTH response, high anti-FML antibodies.	
						
FML	Saponins (Riedel De Haen(R), QuilA, Qs21), IL-12	Swiss Albino	VL/*L. donovani*	Protection	High anti-FML IgG1, IgG2a, and IgG2b, positive DTH response, 73%, 93%, and 79.2% liver parasite reduction in R-FML, QuilA-FML, and Qs21-FML vaccinees respectively, high IFN-γ level in QS21-FML and R-FML vaccines	
						
FML	Fractions of Riedel De Haen—QS21 and deacylsaponins	Swiss Albino	VL/*L. chagasi*	Protection	95% and 86% liver parasite reduction in QS21-FML and deacylsaponins-FML vaccinees respectively, positive DTH response, high IFN-γ production, high IgG, IgG1, IgG2a, IgG2b, and IgG3 in QS21-FML vaccinees but not in deacylsaponins	
						
GP36	Saponin	BALB/c	VL/*L. donovani*	Protection	68.1% liver parasite reduction, high IgG2a, IgG2b, and IgG1 antibodies, positive DTH response	
FML	------	Dogs	VL	Protection	92% protection achieved after two years, vaccinees showed positive DTH response.	
FML	QuilA	Dogs	VL	Protection	95% protection achieved, positive DTH response	
						
FML	QuilA	Dogs	VL/*L. donovani*	Protection	60% dogs protected, high anti-FML IgG, IgG2	
						
FML	Saponin	Dogs	VL	Protection	90% dogs protected, 79–95% positive DTH response, high IgG2 than IgG1	
						
FML	Saponin	Dogs	VL	Protection	Highanti-FMLantibodies, 82.7% positive DTH response, increase in CD8^+^ TandCD21^+^ Bcells	
						
FML	Saponin	Dogs	VL	Protection	Act as a transmission-blocking vaccine, high IFN-γ, NO. and IgG2 production, high CD8^+^ T cell proliferation	^165^^-^^168^
						
LiESA	MDP	Dogs	VL/*L. infantum*	Protection	92% vaccine efﬁcacy, high IgG2 level, enhanced IFN-γ and no production while no change in IL-4 level	^169^^,^^170^
						
LiESA	MDP	Dogs	VL/*L. infantum*	Protection	Increased IFN-γ and anti-LiESA IgG2. level, positive DTH response	
						
Recombinant CP (rCP5)	IL-12	C57BL/6	CL/*L. mexicana*	Protection	-----	
						
CP	CFA	BALB/c	CL/*L. major*	Protection	Enhanced splenocyte proliferation and IFN-γ level, no IL-5 production.	
						
rCPA rCPB	Poloxamer 407	BALB/c	CL/*L. major*	Partial protection	Only by rCPB, enhanced IFN-γ level, equal IgG1, and IgG2a antibody levels	
						
rCPA/rCPB	Fused hybrid in pET23a	BALB/c	CL/*L. major*	Partial protection	High IgG2a, enhanced IFN-γ production with little IL-5	
						
Peptide I of CP	----	CBA	CL/*L. amazonensis*	Protection	Enhanced IFN-γ, IL-4, and NO production, Proliferation of CD8^+^ T-cell subsets	
rGRP78	CFA	C57BL/6	CL/*L. major*	Protection	83% mice protected.	
						
78 kDa	-----	BALB/c	VL/*L. donovani*	-----	Increase in IgG2a levels, low IgG1	
						
78 kDa	MPL-A, liposomal encapsulation, rIL-12, ALD, CFA	BALB/c	VL/*L. donovani*	Protection	92%, 93.4%, and 98% liver parasite reduction by 78 kDa+MPL-A or liposomal encapsulation or rIL-12 vaccinees, enhanced IFN-γ and IL-2 levels with low IL-4 and IL-10, positive DTH response, high IgG2a level	
						
P4 P8 A2	*C. parvum*	BALB/c	CL/*L. pifanoi/L. amazonensis*	Protection	Only P4 and P8 gave protection and P8 gave cross-protection, high IFN-γ level while no change in IL-2 level	
						
P4	*P. acnes*	BALB/c	CL/*L. pifanoi*	Protection	CD4^+^ T-cell related protection, high IFN-γ, MIF, TNF-α mRNA expression, high IL-2 level, and no change in IL-4 level	
P8	------	Dogs	VL/*L. infantum*	----	High IFN-γ and TNF-α expression in P8-stimulated PBMC, low IL-4 but no IL-10 level	
						
P4 P8	----	Human	CL	----	Enhanced IFN-γ and IL-2 levels in respective antigen-stimulated PBMC culture, extremely low IL-4 level	
						
P4	----	Human	CL	-----	Enhanced IFN-γ level in P4-stimulated PBMC culture, IL-4 detectable	
						
rA2	*P. acnes*	BALB/c	VL/*L. donovani*	Protection	89% liver parasite reduction, enhanced IFN-γ level, no change in IL-4 level, high IgG1, IgG2a, IgG2b, and IgG3	
						
rA2	------	BALB/c	VL/*L. chagasi*	Protection	High IFN-γ production, enhanced CTL activity mediated by CD8^+^ T cells, low antibody response	
						
rA2	Saponin	Dogs	VL/*L. chagasi*	Partial protection	Enhanced IFN-γ while low IL-10 production, increased IgG and IgG2 but not IgG1	
						
rHASPB1	IL-12	BALB/c	VL/*L. donovani*	Protection	91% liver and 70–90% splenic parasite reduction in rHASPB1 vaccinees, increased IL-12 production by DC, exclusive IgG1 response, increased IFN-γ producing CD8^+^ T cells.	
rHASPB1	Montanide	Dogs	VL/*L. infantum*	Partial protection	50% dogs asymptomatic, high anti-HASPB1 antibody titer.	
rLcr1	CFA Ribi adjuvant	BALB/c C3H	VL/*L. chagasi*	Partial protection	In infected mice, high IFN-γ production in both mice, detectable IL-10 but not IL-5 levels in splenocytes to Lcr1 stimulation.	
rLcr1	BCG expressing Lcr1	BALB/c	VL/*L. chagasi*	Protection	High IFN-γ and reduced IL-10 production, no detectable IL-4.	
						
rH1	Montanide	Monkeys	CL/*L. major*	Partial protection	High antibody levels, positive DTH response.	
						
rH1 peptides of H	IL-12 IFA	BALB/c	CL/*L. major*	Partial protection	Partial protection even in absence of adjuvants, LP1-3 also gave partial protection.	
						
rORFF	CFA	BALB/c	VL/*L. donovani*	Partial protection	Detectable anti-ORFF antibody titer, the proliferation of spleen cells	
						
rORFF	CpG ODN	BALB/c	VL/*L. donovani*	Protection	84% liver parasite reduction, enhanced IFN-γ and IgG2a production, NO production dose-dependent.	
						
rORFF	----	BALB/c	VL/*L. donovani*	Partial protection	45–60% parasite reduction, low IgG2a/IgG1 ratio, high IFN-γ, and IL-12 as compared to controls.	
						
rORFF	IL-12 DNA	BALB/c	VL/*L. donovani*	Protection	82% parasite reduction, enhanced IFN-γ, IL-12, and IgG2a production, no change in IL-4 level, enhanced splenocyte proliferation.	
						
rLiP0	CpG ODN	C57BL/6 BALB/c	CL/*L. major*	Protection	Complete protection only in C57BL/6 mice, partial in BALB/c, 150-fold parasite reduction, high IFN-γ, and IgG2a production	
Ribosomal proteins (LRP)	CpG ODN	BALB/c C57BL/6	VL/*L. major*	Protection	Protection in both strains, 3 fold parasite reduction, high IFN-γ level and IgG2a/IgG1 ratio, no increase in IL-4, detectable IL-10	
rKMP-11	ts-mutant expressing KMP-11	BALB/c	CL/*L. major*	Partial protection	-----	
rKMP-11	Hybrid cell vaccine	BALB/c	VL/*L. donovani*	Protection	Enhanced IFN-γ, IL-4, and IL-13 expression but not IL-10	
						
rPFR-2	FIA	Hamster	CL/*L. panamensis/L. mexicana*	Protection	Only female hamster protected against *L. panamensis*, positive DTH response, no protection against* L. Mexicana*	
						
Protein Q	BCG	Dogs	VL/*L. infantum*	Protection	90% protection, positive DTH response,	
						
Protein Q	CpG ODN	BALB/c	VL/*L. infantum*	Protection	99% reduction in liver and splenic parasite burden, high IgG2a/IgG1 ratio, high IFN-γ with low IL-4 production	
						
rTSA	IL-12	BALB/c	CL/*L. major*	Protection	Protection only in rTSA-IL12 vaccinees, induce human PBMC proliferation.	
						
TSA LmSTI1 TSA+LmSTI1	IL-12	BALB/c	CL/*L. major*	Protection	The protection conferred in all three vaccinees group when adjuvant is used, signiﬁcant protection by LmSTI1 + IL-12 and TSA + LmSTI1 + IL-12, partial by TSA + IL-12	
						
TSA+LmSTI1	rhIL-12 + alum	Monkeys	CL/*L. major*	Protection	No lesion development even on rechallenge after 4 months of ﬁrst challenge.	
						
rLMSTI1	Encapsulation in liposomes	BALB/c	CL/*L. major*	Protection	High IgG level and IgG2a/IgG1 ratio	
						
rLMSTI1	Encapsulation of antigen with CpG-ODN	BALB/c	CL/*L. major*	Protection	High IgG titer and IgG2a/IgG1 ratio	
						
rLeish-111f	MPL-SE	BALB/c	CL/*L. major*	Protection	Enhanced IFN-γ and IgG2a production, low IL-4 level	
rLeish-111f	MPL-SE rmIL-12	BALB/c	CL/*L. major*	Protection	Enhanced IFN-γ production, no detectable IL-4, mixed IgG1, and IgG2a response	
						
rLeish-111f	MPL-SE	BALB/c C57BL/6 Syrian hamster	VL/*L. infantum*	Protection	91.7% and 99.6% splenic parasite reduction in mice and hamster respectively, enhanced IFN-γ, IL-2, TNF production with low IL-4 level in mice	
TSA+LmSTI1 + LeIF+Lbhsp83	GM-CSF	Human	MCL	Protection	83% of patients showed complete clinical cure (CC) after nine months, all were CC after a five-year follow-up	
rTSA rLeIF rLbSTI1 rLACK	CpG ODN	BALB/c	CL/*L. braziliensis*	No Protection	Enhanced IFN-γ production in response to TSA or LeIF or LACK stimulation, high IgG1/IgG2a ratio	
rTSA+rLeIF+ rLmSTI1	MPL-SE AdjuPrime	Dogs	VL/*L. chagasi*	-----	Induce Th1 response, speciﬁc IgG response to all three antigens, high IgG2a/IgG1 ratio when MPL-SE is used as compared to AdjuPrime	
rMML	MPL-SE AdjuPrime	Dogs	VL/*L. infantum*	No protection	87% cumulative incidence in vaccines even after two years of vaccination	
rLeish-110f+ Glucantime	MPL-SE	Dogs	VL/*L. chagasi*	protection	83.3% and 66.6% survival rate by immunochemotherapy and chemotherapy respectively, high proliferative response, high antibody titer in immunotherapy as compared to immunochemotherapy	

**Third-generation vaccines**

***DNA vaccines***

These vaccines contain plasmid DNA, which, after injection, encodes foreign proteins, leading to the synthesis of endogenous proteins and the production of specific immune responses^[^^84^^]^. DNA vaccines promote both cellular and humoral immunity^[^^85^^,^^86^^]^_. _DNA vaccines can come in many forms, including recombinant proteins^[^^87^^-^^97^^]^, single vaccines^[^^89^^,^^90^^,^^93^^,^^96^^,^^98^^-^^100^^]^, or multigene forms^[^^92^^-^^95^^,^^101^^]^. These vaccines were tested in mice against CL and VL^[^^84^^,^^85^^,^^86^^,^^91^^,^^94^^,^^95^^,^^99^^,^^101^^]^, in hamsters against VL^[^^102^^,^^103^^]^, and dogs against VL^[^^104^^-^^107^^]^. DNA vaccines are made up of heterologous DNA (usually a plasmid) that produces antigenic proteins. These DNAs are supplied by vectors that allow them to be expressed in eukaryotic cells^[^^84^^]^. Advantages of DNA vaccines include (1) fast, simple, and cheap large-scale production, (2) no need for low temperature, transportation, and storage, and (3) the ability to provide long-term protection against multiple strains of *Leishmania*. The main concern with these vaccines is the risk of parasite DNA entering the mammalian genome. This problem carries the potential risk of cancer and autoimmune diseases^[^^84^^]^. A summary of DNA vaccines is given in Table 6 and the best recombinant salivary candidates is shown in Table 7.

**Vaccine products for potential licensing**

There are no licensed products yet, but potential candidates could be as follows^[^^108^^]^: (1) a mixture of recombinant proteins (Leish F1, Leish F2, and Leish F3), designed by Infectious Disease Research Institute (Seattle, USA), is currently in the second phase of a clinical trial; (2) recombinant proteins from *Leishmania* and sandfly saliva (*phlebotomus*) antigens, designed by Sabin product development partnership (Washington, USA)^[^^19^^]^, is now in the preclinical phase. FML-QuilA (Leishmune®), a protein vaccine, was the first approved vaccine in Brazil in 2003. However, the license to produce and sell the vaccine was suspended in 2014, and its production was stopped by factories. The reason for discontinuation was the incompleteness of the third phase of the trial. There are presently two vaccines against canine VL: A2 Leishmanial Ag from Brazil and Li ESP/QA-21 from France^[^^19^^]^.

## Discussion

Vaccines are undoubtedly the most effective way to control diseases. For this reason, the development of safe and cost-effective vaccines, particularly for the diseases with no available vaccine (e.g. leishmaniasis) is an important global public health priority. A major barrier to the development of an effective vaccine is related to the discrepancies between the animal models and human diseases, as well as the transition of the research from the laboratory to the field. Additionally, many questions related to the immune responses and maintenance of immunological memory during an active *Leishmania* infection have not yet been extensively studied or answered. This article tried to focuse on the latest information related to antileishmanial vaccine development and also major problems with vaccine development and implementation. Candidates for the *Leishmania* vaccines include leishmanization, as well as the first-, second-, and third-generation vaccines. The development of an effective *Leishmania* vaccine poses many challenges, mainly related to the complexity of the immune responses to *Leishmania*, insufficient knowledge of *Leishmania* pathogenesis, and the discrepancy between the Old and New World parasites. It appears that a successful vaccine will most likely be composed of several antigens rather than a single one, which suggests that combination vaccines and well-developed adjuvants, such as Leish-111f and MPL-SE, have the best chances of success. Further clinical trials provide more information on the success of these combination vaccines. In addition, the poor efficacy of the killed and subunit vaccines makes the use of live-attenuated vaccines the next best alternative^[^^109^^]^. Many questions about antileishmanial immunity in humans have not yet been answered. It is not clear whether parasite persistence is required to maintain immunity in humans. Although parasite persistence in humans is unknown, it is worth noting that an experimental mouse model has revealed the persistence of the parasite following infection^[^^110^^]^. A study has been shown that the absence of parasites leads to the loss of immunity, implying that continuous antigen presence is needed for complete protection^[^^22^^]^. 

In contrast, another study in a mouse model has revealed that the maintenance of memory T-cells is independent of parasite persistence, and therefore vaccination with non-persistent strains and non-persistent, attenuated strains such as LdCEN^-/-^ or ∆PMM results in long-term protection^[^^22^^]^. In general, due to the complex nature of the immune response to *Leishmania*, it is crucial to better understand the determinants of T-cell for long-term immunity and the immunity factors affecting antileishmanial immunity before the development of an effective vaccine. Our understanding of the determinants of T cells is required for long-term protective immunity, although there are still many unknowns. It is hoped that new strategies will be developed to produce effective T-cell vaccines. 

**Table 6:** Third-generation vaccines against *Leishmania*

**Antigen**	**Adjuvant/delivery system**	**system** **Animal model**	**Targeted disease (** ***Leishmania*** ** spp.)**		**Result**		**Other outcomes**	**Ref.**
gp63	pCMV	BALB/c	CL/*L. major*		Protection		Enhanced IL-12 and IFN-γ production, no detectable IL-4	^90, 214^
						
gp63	pCMV3ISS or pcDNA3	BALB/c	CL/*L. major*		Partial protection		30% of mice protected, enhanced IFN-γ protection but not IL-4	^94, 215^
						
gp63 or gp46	VR1012	BALB/c	CL/*L. mexicana*		Partial protection		100-fold parasite and 30% reduction in lesion size, mixed IgG2a and IgG1 response, high IgG2a/IgG1 in gp46 vaccinee	^216,217^
						
gp63 + gp46 + CPb	VR1012	BALB/c	CL/*L. mexicana*		Protection		80% and 1,000-fold reduction in lesion size and parasite burden respectively	^216,217^
						
ORFF	pcDNA3.1	BALB/c	VL/*L. donovani*		Protection		78–80% and 58–60% reduction in liver and spleen parasites respectively, enhanced IFN-γ expression but no change in IL-4 expression	^99^
						
PSA-2	pCI-neo	C3H/He	CL/*L. major*		Protection		Enhanced IFN-γ production as compared to control, no detectable IL-4 and IL-5, high IgG2a/IgG1 ratio	^218,219^
						
A2	pcDNA3	BALB/c	CL/VL *L. amazonensis/L. chagasi*		Protection		Protection against both species enhanced IFN-γ with low IL-4 and IL-10 production	^220^
						
LACK	pCI-neo	BALB/c	VL/*L. chagasi*		Protection		Increased IFN-γ and IL-4 production with low IL-10 and TNF-α level	^221^
						
LACK	pCI-neo	BALB/c	VL/*L. chagasi*		No protection		Increased IFN-γ and IL-10 production with no IL-4	^222^
						
LACK	pCI-neo	BALB/c	VL/*L. chagasi*		No protection		Enhanced IFN-γ with no IL-4 production	^223^
						
LACK	pCMV3ISS	BALB/c	CL/*L. major*		Partial to complete protection		Partial protection by LACK vaccine while complete in LACKp24 vaccinees	^94^
						
LACK	MIDGE or MIDGE-NLS	BALB/c	CL/*L. major*		Protection		Enhanced IFN-γ production with no IL-4, high IgG2a/IgG1 ratio	^224^
CPa or CPb CPa + CPb	pCB6	BALB/c	CL/*L. major*		Protection		Protection only in CPa + CPb vaccines increased IFN-γ level, but no IL-5	^92^
						
CPb	VR1012	BALB/c	CL/*L. mexicana*		Partial protection		100-fold parasite and 50% reduction in lesion size	^216,217^
						
KMP-11	pCMV-LIC	Hamster	VL/*L. donovani*		Protection		Inducemixed Th1/Th2 response, enhanced IFN-γ, TNF-α, IL-12, iNOS expression including IL-4, low IL-10 level, high IgG2a, and IgG1 titer	^225^
						
KMP-11	pCMV-LIC	BALB/c	VL/*L. donovani*		Protection		96.7% and 98.7% reduction in splenic and liver parasite respectively, enhanced IFN-γ and IL-4 production, suppressed IL-10 level	^226^
						
KMP-11	pCMV-LIC+IL-12	BALB/c	CL/*L. major*		Protection		93% reduction in lesion size, enhanced IFN-γ with suppressed IL-4 and IL-10 production	^226^
						
P4	pcDNA3+IL-12 or HSP70	BALB/c	CL/*L. amazonensis*		Partial to complete protection		Complete protection with enhanced IFN-γ and TNF-α, low IL-10 production in P4 + IL-12 vaccines while partial with mixed IFN-γ and IL-10 response in P4 + HSP70 vaccines	^101^
						
NH36	VR1012	BALB/c	VL/*L. chagasi*		Protection		91% liver parasite reduction, increased IFN-γ with reduced IL-10 and IL-4 levels, positive DTH response, high IgG2b titer	^227^
						
papLe22	pcDNA3.1	Hamster	VL/*L. infantum*		Partial protection		Parasite circulation reduced by 50%, produce high anti-pepLe22 but low anti-*Leishmania *antibody titer	^91^
						
NH	VR1012	BALB/c	CL/VL *L. amazonensis/L. chagasi*		No protection		Enhanced IFN-γ, IL-4, and IL-10 production	^228^
						
NH36	VR1012	BALB/c	CL/VL* L. chagasi/L. mexicana*		Protection		88% and 65% reduction in *L. chagasi* parasite burden and *L. mexicana* infected lesion size respectively, 2–5 fold increase in IFN-γ producing CD4^+^ T cells, low antibody response, positive DTH response to *L. donovani*	^96^
						
LeIF PSA-2	pCMV3ISS	BALB/c	CL/*L. major*		No protection		---------	^94^
						
TSA LmSTI1 TSA+LmSTI	pcDNA3	BALB/c	CL/L*. major*		Protection		Protection induced by all three vaccines enhanced IFN-α production with no IL-4, high IgG2a titer	^93^
						
H2A+H2B+ H3+H4	pcDNA3	BALB/c	CL/*L. major*		Protection		Enhanced IFN-γ with little IL-4 production, low antibody response dominated by IgG2a	^95^
						
KMPII+TRYP+ LACK+gp63	pMOK	Dogs	VL/*L. infantum*		No protection		Increased anti-*Leishmania* IgG, IgA, and IgM	^97^
						
LACK-PB	pcDNA3-vaccinia virus	BALB/c	CL/*L. major*		Protection		1,000 fold and 70% decrease in parasite burden and lesion size respectively, increased IFN-γ level with low IL-10 and IL-4 levels	^229^
**Heterologous prime-boost vaccine**								
LACK-PB	pCI-neo—vaccinia virus	Dogs	VL/*L. infantum*	Protection		60% of dogs protected, enhanced IFN-γ and IL-4 expression, high IgG2a/IgG1 ratio		^106^
						
LACK-PB	pcDNA3.1 + IL-12 DNA or IL-18 DNA—vaccinia virus	BALB/c	CL/*L. major*	Protection		Enhanced IFN-γ production, high IgG2a/IgG1 ratio		^230^
						
LACK-PB	pCI-neo—MVA	BALB/c	CL/*L. major*	Protection		65–92% reduction in lesion size, increased IFN-γ and TNF-α levels		^231^
						
LACK-PB	MVA	BALB/c	VL/*L. infantum*	Protection		144–244, 6–9, and 9–30 fold parasite reduction in the lymph node, spleen, and liver respectively, increased IFN-γ and TNF-α levels		^232^
						
LACK-PB	pcDNA3—*Salmonella enterica* serovar *Typhimurium*	BALB/c	CL/*L. major*	Protection		Increased IFN-γ level with low IL-10, high IgG2a titer		^233^
LACK-PB	pCI-neo—MVA	Dogs	VL/*L. infantum*	Protection		Increased IFN-γ expression with low IL-10 and IL-4 transcripts, high IgG2 titer		^220^
						
CPa+CPb-PB	pCB6 + CpG ODN + Montanide 720	Dogs	VL/*L. infantum*	Protection		High IFN-γ/IL-10 ratio, increased IgG, IgG2 but not IgG1 titer, positive DTH response		^107^
						
CPa+CPb-PB	pCB6 + CpG ODN + Montanide 720	BALB/c	VL/*L. infantum*	Protection		Increased IFN-γ, high IFN-γ/IL-5 ratio, high IgG and IgG2a titer, low IgG1		^234^
						
CTE of CPb-PB	CpG ODN + Montanide 720	BALB/c	VL/*L. infantum*	No protection		Increased IL-5 level, high IL-5/IFN-γ ratio, high IgG2a/IgG1 ratio		^235^
						
CPc-PB	pcDNA3.1 + DHFR + CpG ODN+ Montanide 720	BALB/c	VL/*L. infantum*	Protection		Enhanced IFN-γ and NO production, high IgG2a/IgG1 ratio		^236^
						
LiP0-PB	pcDNA3	BALB/c	CL/*L. major*	Protection		84.8–99.1% parasite reduction, enhanced IFN-γ production, mixed IgG2a/IgG1 response		^237^

**Table 7:** The best recombinant salivary candidates as antigens for detection of anti-saliva antibodies

**Recombinant** **protein**	**Protein** **family**	**Sandfly species**	**Host** **species**	**Reference**
LJM17	YRP	*Lu. longipalpis*	dog, fox, human	^238,239^
LJM11	YRP	*Lu*. *longipalpis*	human, dog, chicken	^238,240^
LJM17+LJM11	YRP	*Lu*. *longipalpis*	human	^238^
rPpSP32	SP32-like	*Phlebotomus papatasi*	human	^241-243^
rPorSP24	YRP	*P*. *orientalis*	sheep, goat, dog	^244^
rSP03B	YRP	*P*. *perniciosus*	mouse, dog, hare, rabbit	^245-248^
rSP01	apyrase	*P*. *perniciosus*	mouse, dog	^245^
rSP01B	apyrase	*P*. *perniciosus*	mouse, dog, hare, rabbit	^245,246,249^

*Lu*. Longipalpis, *Lutzomyia* *longipalpis*

The most important thing to consider before making a *Leishmania* vaccine is to determine the best immunity correlations, as well as to develop efficient delivery systems and improved adjuvants. According to advanced research in parasite immunology and genetic engineering, an effective anti-*Leishmania* vaccine not far away. In this study, data extraction was performed by two researchers, which may result in errors. Searching for English language and scientific articles in other languages, which may have valuable information from Africa, the Middle East, and Asia, were limited. Despite these limitations, the present study attempted to review the content of credible articles that lead to clear and up-to-date information on the performance and effectiveness of various vaccines designed against leishmaniasis. 

Given the global importance of leishmaniasis, decisive measures must be taken to prevent this disease with social impacts. It seems that one of the effective ways to control leishmaniasis is immunization of people living in endemic areas of the disease. In this review, it was found that an effective vaccine against leishmaniasis is not yet available, and scientists in this field have chosen different methods to produce such a vaccine. The results of these efforts have been the production of three different generations of *Leishmania* vaccines. In any case, summarizing the results of these studies and trying to clarify as much as possible the ambiguities in the immunity of leishmaniasis and especially the interaction of the parasite with host cells will help to advance in the right direction. Understanding more about the unknown mechanisms of the behavior of the parasites inside the host body will persuade us to produce an effective vaccine against the disease.

## CONFLICT OF INTEREST.

None declared.
